# Docosahexaenoic Acid and Cognition throughout the Lifespan

**DOI:** 10.3390/nu8020099

**Published:** 2016-02-17

**Authors:** Michael J. Weiser, Christopher M. Butt, M. Hasan Mohajeri

**Affiliations:** 1 DSM Nutritional Products, R&D Human Nutrition and Health, Boulder, CO, USA; Chris.Butt@DSM.com; 2 DSM Nutritional Products, R&D Human Nutrition and Health, Basel, Switzerland; hasan.mohajeri@dsm.com

**Keywords:** brain lipids, omega-3 polyunsaturated fatty acids, nutrition, learning, memory, comprehension, development, aging, neurodegeneration

## Abstract

Docosahexaenoic acid (DHA) is the predominant omega-3 (*n*-3) polyunsaturated fatty acid (PUFA) found in the brain and can affect neurological function by modulating signal transduction pathways, neurotransmission, neurogenesis, myelination, membrane receptor function, synaptic plasticity, neuroinflammation, membrane integrity and membrane organization. DHA is rapidly accumulated in the brain during gestation and early infancy, and the availability of DHA via transfer from maternal stores impacts the degree of DHA incorporation into neural tissues. The consumption of DHA leads to many positive physiological and behavioral effects, including those on cognition. Advanced cognitive function is uniquely human, and the optimal development and aging of cognitive abilities has profound impacts on quality of life, productivity, and advancement of society in general. However, the modern diet typically lacks appreciable amounts of DHA. Therefore, in modern populations, maintaining optimal levels of DHA in the brain throughout the lifespan likely requires obtaining preformed DHA via dietary or supplemental sources. In this review, we examine the role of DHA in optimal cognition during development, adulthood, and aging with a focus on human evidence and putative mechanisms of action.

## 1. Introduction

The cognitive ability of humans is arguably the most advanced in the entire animal kingdom. Such advancement is believed to be conferred by an expanded cerebral cortex and a highly developed prefrontal cortex, both of which are brain regions important for cognition. There are many different domains to the clinical construct of cognition. These include attention, memory (working and long-term), perception, language, problem solving, comprehension, reasoning, computation, reading and speech. Cognition changes throughout the lifespan matching the development, maturation and aging of the brain. The brain is a lipid-rich organ that consumes 20% of the body’s energy, but it only comprises 2% of the body’s mass. Over half of the brain’s dry weight is comprised of lipids, and it is especially enriched in long-chain omega-3 (*n*-3) polyunsaturated fatty acids (PUFAs), suggesting a key role for these molecules in the optimal development, maturation and aging of neural structures and networks. A substantial amount of literature exists that highlights the crucial role of nutrition in brain development, and thus on brain function and mental performance in humans. Ultimately, the proper functioning of the brain has significant dependence upon maintaining its optimal lipid composition [1].

Quantitatively, docosahexaenoic acid (DHA; 22:6*n*-3) is the most significant *n*-3 PUFA in the brain as both eicosapentaenoic acid (EPA; 20:5*n*-3) and α-linolenic acid (ALA; 18:3*n*-3) are present in only very small quantities. DHA makes up over 90% of the *n*-3 PUFAs in the brain and 10%–20% of its total lipids. DHA is especially concentrated in the gray matter [2]. It is stored primarily in phosphatidylethanolamine (PE) and phosphatidylserine (PS) membrane phospholipids, with smaller amounts also found in phosphatidylcholine (PC; [3]), where it plays an important role in the biosynthesis of PS (DHA-PS) in the brain [4]. DHA is enriched in membranes structures found at synaptic terminals, mitochondria and endoplasmic reticulum [5], and it can ultimately affect cellular characteristics and physiological processes including membrane fluidity, lipid raft function, neurotransmitter release, transmembrane receptor function, gene expression, signal transduction, myelination, neuroinflammation, and neuronal differentiation and growth [6,7,8].

The brain’s frontal lobes are particularly responsive to the supply of DHA during development [9]. Decades of work have clearly established the responsibilities of the frontal lobes for executive and higher-order cognitive activities including sustained attention, planning and problem solving [10], and the prefrontal lobe in particular for social, emotional and behavioral development [11]. Therefore, maintaining optimal lipid composition in these brain regions, and specifically DHA levels, is not only important during the development and maturation of the brain from gestation through childhood and adolescence [12,13], but such maintenance is also critical for successful aging of the adult brain [1,14,15,16]. In this review, we will discuss the importance of DHA for optimal neurological health throughout the lifespan, with a particular emphasis on cognitive function.

## 2. DHA Delivery to the Brain

DHA synthesized *de novo* originates from ALA via a series of desaturations and elongations primarily within the endoplasmic reticulum (ER), with the exception of the last step, a β-oxidation from tetracosahexaenoic acid (24:6*n*-3) that occurs in peroxisomes [17]. ALA is considered an essential nutrient because humans lack the *n*-3 desaturase enzyme required for its production. However, it could be argued that DHA is also an essential nutrient due to inefficiencies of the 5-desaturase and 6-desaturase enzymes (FADS1/2) needed for its biosynthesis, and the competition for these enzymes by the omega-6 (*n*-6) PUFA linoleic acid (LA; 18:2*n*-6). LA is typically consumed in high amounts in modern diets, which exacerbates the increase of *n*-6 PUFAs, as well as the decrease of *n*-3 PUFAs, that are incorporated in peripheral and neural tissues [18]. Therefore, many researchers conclude that preformed DHA consumption is required for reaching and maintaining ideal brain DHA concentrations and related neurological functions [19,20,21].

There is a general consensus on the recommended daily average intake requirements for infants. This consensus is driven by data from randomized control trials (RCTs) that suggest a minimum level of DHA at 0.32% of total fatty acids in formula [22,23,24], which is towards the lower half of the global concentration range found in human milk (0.06% to 1.4% of total fatty acids) [25,26]. The evidence for daily intake requirements for children and adults is still emerging. Nonetheless, current guidelines are in the range of 250 to 500 mg EPA + DHA per day [27,28,29]. However, most people do not consume enough *n*-3 PUFAs, as indicated by average modern daily dietary DHA intakes that are closer to 100 mg per day [21,30,31].

The shift in modern diets towards reduced *n*-3 PUFA intake, increased *n*-6 PUFA consumption, combined with less physical activity has had a detrimental impact on development and aging, especially with regard to cognitive function. The optimal dietary *n*-6 to *n*-3 PUFA ratio is 2:1 and below, while the current Western diet is typically in the range of 10:1 to 25:1 [32]. DHA likely played an important role in the evolution of the human brain, as significant structural changes in the encephalon and gains in cognitive abilities coincided with the use of aquaculture as a substantial portion of the human diet [33,34]. Habitats near aquatic food sources, such as lakes, rivers, and the sea provided rich dietary sources of DHA. It therefore follows that sources of preformed DHA include oils from microalgae, fatty fish (especially salmon, mackerel, sardines, and herring), and fish oil.

The human brain metabolizes approximately 4 mg of DHA per day, resulting in an estimated half-life of brain DHA of 2.5 years [35], much longer than that of DHA in peripheral tissues (e.g., two minutes in plasma; [35]). Importantly, although EPA does have significant acute anti-inflammatory actions in neural tissue [36,37] and the absorbance of EPA and DHA to the brain are similar [38,39], EPA levels are extremely low in brain tissue. This circumstance occurs because EPA is rapidly oxidized and removed from the brain, or it is elongated to the *n*-3 PUFA docosapentaenoic acid (DPAn3; 22:5*n*-3), which acts as a precursor for DHA [40]. However, EPA conversion is not a significant source of DHA [41,42,43]. The rapid β-oxidation of EPA may be the result of increased uptake of EPA by peroxisomes, where long chain fatty acids are initially catabolized before undergoing mitochondrial oxidation [44,45]. Such energetically costly mechanisms suggest a unique requirement for DHA. Therefore, for the purposes of this review we will consider the effects of combined *n*-3 PUFA (DHA + EPA) consumption or supplementation on brain function and cognition as being significantly dependent on DHA.

DHA is acquired during development through gestational placental transfer and from mother’s milk during infancy. The levels of DHA in mother’s milk can be as high as 1.4% of total fatty acids with a worldwide average of 0.32% of total fatty acids, depending on the mother’s diet and number of pregnancies [25,46]. *De novo* DHA synthesis can occur in the liver, however the DHA precursor ALA does not increase plasma DHA in humans (<0.1% conversion efficiency in humans; [47,48,49]). In fact, neither ALA nor EPA is an effective dietary source of DHA due to the minimal *in vivo* production of DHA from these precursors in humans, indicating that preformed DHA is most effective in maintaining sufficient tissue stores [20]. On the other hand, DHA supplementation adds to the peripheral tissue pool of EPA, likely due to retroconversion [50].

DHA accretion in the brain accelerates during the middle of gestation, slows down in infancy, and reaches a plateau in early adulthood [51,52]. Half of the brain’s DHA is accumulated during gestation, and the infant brain acquires five-times the level of lipids on a daily basis as the adult brain [53,54]. In adults, accretion is slower, and individuals with red blood cell DHA concentrations on par with the average European or American (~4% of total fatty acids) require 4–6 months of oral DHA supplementation to reach a steady state concentration that is dependent on DHA dose (8%–9% for 1000 mg per day; 5%–6% for 200 mg per day) [41]. Local *de novo* DHA synthesis in the brain is very low, thus DHA levels are maintained via delivery from the blood [55,56]. Circulating levels of DHA in the blood can reach as high as ~5% of that which is ingested orally [57] and ~0.5% of the circulating level is delivered to the central nervous system (CNS; [35]). After oral ingestion of DHA, lipases in the gut deliver unesterified free fatty acid (DHA-FFA) to the small intestine, and processing by the small intestine and liver results in circulating versions of DHA as DHA-triacylglycerides (DHA-TAGs), DHA-PC and DHA-FFA bound to low density lipoprotein (LDL) and albumin. These various forms are dissociated at the blood-brain barrier (BBB) through both active and passive processes that are mediated by endothelial lipases, fatty acid binding proteins (FABPs), and apolipoprotein E (ApoE) [58,59,60,61]. Unesterified DHA freely passes the BBB [39,61], and it appears that the brain derives most of its DHA from the unesterified FFA pool in blood [55]. Within the central nervous system, DHA is transported primarily via FABPs [59,60] and ApoE produced by astrocytes [61]. Membrane-incorporated DHA cycles in and out of the membrane from the phospholipids to the intracellular FFA pool via actions of DHA-coenzyme A (DHA-CoA) [62,63], providing a mechanism to respond to dynamic cellular events and to challenges during development and aging.

## 3. DHA and Cognition in Development

### 3.1. DHA during Gestation and Infancy

DHA is necessary for the growth and maturation of an infant’s brain and retina [26,64,65], and as outlined earlier, DHA is conditionally essential for humans since it cannot be synthesized efficiently. This particular point needs to be stressed in that when DHA is needed in large amounts, such as during rapid phases of brain growth, it needs to be consumed via external sources. Furthermore, synthesis of DHA from its precursors in the fetus and placenta is insufficient to meet the demand of rapidly developing neural tissues [26,66,67,68], requiring the delivery of maternal DHA stores via placental transfer and mother’s milk during pregnancy and after birth, respectively [66,67]. Therefore, the adequate supply of DHA to the developing brain is largely dependent on the dietary intake of the mother [69], and this supply is very important to the cognitive development of the progeny [70,71].

The observations above have led to the recommendation that pregnant and nursing women should consume at least 200 mg of DHA daily [26,72]. Supplementation with DHA during pregnancy may also benefit both the mother and baby by extending the length of gestation. A Cochrane review in 2006 concluded that maternal *n*-3 PUFA supplementation during pregnancy increased the gestational age by 2.5 days resulting in an average increase of birth weight by 50 g and birth length by 0.5 cm [73]. In alignment with this finding, a meta-analysis revealed that maternal *n*-3 PUFA supplementation was associated with pregnancies averaging 1.6 to 2.6 days closer to term [74]. A further meta-analysis of six RCTs found a 31% and 61% overall reduction of the risk of preterm births (defined as delivery before the 34th week of gestation) in all pregnancies and high-risk pregnancies, respectively, when mothers supplemented with *n*-3 PUFAs [75]. Lastly, maternal supplementation of 400 mg DHA per day in the second half of gestation was beneficial to children’s growth tested at 18 months of age. This effect was only seen in children born to first time mothers [76]. These effects on growth and development of the fetus likely have a profound impact on cognitive abilities later in life.

Epidemiological studies have shown the importance of DHA during pregnancy for neuronal development. A large study (*N* = 11,875) showed that a lower intake of seafood, a rich source of DHA, during pregnancy was associated with risk of suboptimal development. In contrast, children born to mothers with a high intake of seafood during pregnancy exhibited greater pro-social behavior, better fine motor and social development scores, and higher verbal intelligence at eight years of age [77]. RCT trials have also provided evidence for a positive effect of DHA supplementation during pregnancy. For instance, taking 200 mg DHA orally per day for four months during pregnancy improved cognitive abilities of children tested at five years of age [78]. In a more recent multicenter RCT, 2399 pregnant women, who were <21 weeks into gestation with singleton pregnancies, were supplemented with 800 mg DHA and 100 mg EPA until delivery. While there was no effect of supplementation on overall mean cognitive scores in the offspring when measured at 18 months, the resulting data indicated that *n*-3 PUFA supplementation lowered the number of preterm births and low birth weights, resulted in fewer admissions to neonatal intensive care units, and reduced the number of children with cognitive scores indicative of delayed cognitive development [79].

Postnatally, the importance of DHA obtained from mother’s milk for neuronal development has been reported repeatedly [80,81,82,83,84]. High DHA concentrations in breast milk have been associated with several brain-related positive health benefits in infants. These associations include a better ability to adjust to changes in surrounding [85], better mental development [86,87], improved hand-eye coordination [82], better attention scores [78] and memory performance later in life [88]. Nevertheless, some studies report neutral effects of DHA supplementation of lactating mothers on neurodevelopmental outcomes, results which likely depend on length and timing of DHA supplementation as well as the developmental time points assessed [82].

The DHA content of mother’s milk is directly dependent on the mother’s diet [26,89,90]. Dietary intake of DHA leads to a dose-dependent increase in DHA levels of breast milk [64,91]. Typically, DHA levels in tissues are higher in breastfed infants when compared to formula-fed infants [92]. Thus, it is not surprising that breastfeeding in the first six months of life is recommended for the optimal development of the baby, provided that the mother’s nutritional status is favorable [81,93]. This is an important recommendation, and is corroborated by many studies that attest to superior neuronal development of breastfed infants in comparison to infants fed with formula (for a review, see [90]). For example, the Western Australian Pregnancy Cohort (Raine) Study recruited 2900 pregnant women and followed the live births for 14 years. This long-term study provided evidence that a shorter duration of breastfeeding may be a predictor of adverse developmental mental health outcomes throughout childhood and early adolescence [94]. 

PUFA supplementation of infant formula is an effective means to realize the benefits of breast milk in infants who are not able to be breastfed. For example, a 2007 meta-study summarized several RCTs that had examined PUFA supplementation of term infants and found a consistent association of DHA (and ARA) supplementation with beneficial effects on visual development in the first year of life [95]. For example, in a prospective, double-blind RCT (the DIAMOND study), 181 infants were enrolled at 1–9 days of age and assigned randomly to receive one of four term infant formulas with one of four levels of DHA: Control (0% DHA, 0% ARA), 0.32% DHA, 0.64% DHA, or 0.96% DHA for 12 months. All DHA-supplemented formulas contained 0.64% ARA. At 18 months of age, children who received DHA-supplemented formulas showed a significantly higher MDI score [96]. In another trial, 420 healthy term infants were randomized to receive a DHA-enriched fish oil supplement (containing at least 250 mg DHA and 60 mg EPA per day) or a placebo from birth to six months. Developmental assessment occurred at 18 months via the Bayley Scales of Infant and Toddler Development and the Child Behavior Checklist. Language assessment occurred at 12 and 18 months via the Macarthur–Bates Communicative Development Inventory. When compared to placebo, the fish oil group had significantly higher erythrocyte and plasma phospholipid DHA levels at six months of age. In a small subset analysis (about 40% of the total population), children in the DHA-enriched fish oil group had significantly higher percentile ranks of both later developing gestures at 12 and 18 months and the total number of gestures [97]. Supplementation of infant formula with DHA and ARA (0.32% and 0.72% of total fatty acids, respectively) provided for the first 17 weeks of life resulted in visual acuity scores similar to breastfed infants who performed better than control formula fed children at four years of age [98]. However, linolenic acid (precursor to DHA) provided via formula was ineffective in a separate trial in a similar measure of visual acuity at 16 and 34 weeks of age [99]. These results suggest that dietary supplementation of DHA during the first year of life likely leads to enhanced cognitive performance.

The studies cited above highlight the importance of maintaining sufficient intake of DHA during both pregnancy and nursing. For example, Helland *et al.* found that the supplementation of pregnant women with *n*-3 PUFAs from Week 18 of pregnancy through lactation conferred a cognitive benefit to their children on an intelligence test at four years of age, and the cognitive benefits in the children correlated significantly with maternal intake of DHA [87]. Furthermore, mathematical modelling revealed that a 100 mg/day increase in maternal DHA intake resulted in a small but significant increase in the intelligence quotient (IQ) of infants between 10 and 39 months of age [100]. Systematic review of maternal DHA supplementation indicated that DHA supported mental development and longer-term cognitive functioning of the child. When DHA was given to pregnant and lactating women, an increase in maternal intake of 1 g per day of DHA increased the child’s IQ by 0.8 to 1.8 points [100]. However, when pregnant mothers were supplemented with 4 g of fish oil daily for a shorter period (only during late gestation) no effects were observed on overall mental or psychomotor development when measured at 10 months of age, but did positively affect hand and eye coordination at 2.5 years [101,102]. Similarly, supplementation of infant formula with DHA (0.2% of total fatty acids) for the first four months of infancy had no effect on Bayley Mental Index scores at 12 months of age [103]. These data suggest that the best neurodevelopmental effects of DHA, including those on cognitive performance, might be best achieved by exposure during both pregnancy and lactation [87]. Such findings emphasize the urgency of adequate intake of dietary DHA by pregnant and lactating women to ensure the healthy development of the brain and visual system in their offspring.

### 3.2. Preterm Infants and DHA

As alluded to above, the rapid growth of the brain during the last trimester of pregnancy requires a relatively high amount of DHA for proper brain development. Due to this circumstance, infants born prematurely are uniquely susceptible to the effects of DHA deficiency. Moreover, the ability of preterm infants to synthesize DHA *de novo* via elongation and desaturation of precursor fatty acids is insufficient to make up for their substantial deficits in DHA tissue levels [104]. Beneficial effects of PUFA supplementation in preterm babies for cognitive [105] and psychomotor development are well-established [106]. For example, Henriksen and colleagues showed that supplementation of expressed mother milk with 32 mg DHA and 31 mg of the *n*-6 PUFA arachidonic acid (ARA; 20:4*n*-6) per day resulted in better cognitive performance of premature infants weighing 1500 g or less at birth when examined six months later [107].

PUFA supplementation of infant formula for preterm infants has consistently demonstrated positive effects on aspects of neurobehavioral development [108]. High-DHA (approximately 1% total fatty acids) formula was compared to standard DHA (approximately 0.3% total fatty acids) from Day 2 to 4 of life until term-corrected age in an RCT of preterm infants. At 18 months of age, infants born at less than 1250g in the high DHA group had a 50% lower likelihood of showing mental delay, as measured by the Bayley Mental Delay Index (MDI), when compared to infants in the standard DHA group [109]. In addition, an overall positive effect of DHA on MDI measured at 18 months of age was only observed among girls, who had less than half the risk of mild mental delay and less than a fifth of the risk of severe mental delay after high DHA supplementation compared to the standard DHA group. This effect was strongest among infants of mothers with low level of education, while no significant effect on mental development was seen among infants of mothers with higher education. However, when the same group was studied seven years later no differences between high-DHA and standard-DHA groups were evident in measures of weight, height, head circumference, or visual function [110,111]. Overall, current data suggest that infants fed formula benefit greatly from standard DHA supplementation and that this is especially important for preterm infants.

### 3.3. DHA during Infancy and the Relationship to Socioeconomic Conditions

The effect sizes associated with *n*-3 PUFA supplementation on postnatal growth and neurodevelopment in infants are relatively small. This circumstance is likely due to study populations that have been limited to formula-fed infants in high-income countries. Well-designed studies examining the effect of postnatal PUFA supplementation on infant growth in low-income countries are scarce, but some emerging data exist. For instance, a trial in rural Gambia that randomized 183 infants to receive fish oil or olive oil placebo from 3 to 9 months of age resulted in significant increases of growth parameters, such as mid-upper arm circumference and skin thickness in the fish oil group [112]. Interestingly, a recent Cochrane review came to the conclusion that, in high-income countries, “there is inconclusive evidence to support or refute the practice of giving PUFA supplementation to breastfeeding mothers in order to improve neurodevelopment,” a similar conclusion to that of Scholtz *et al.* in a review highlighting the need for better study designs in future studies [113]. Nonetheless, the Cochrane review reported moderate positive evidence for measures obtained beyond 24 months related to language development and body weight in children. However, the authors also reported neutral findings for outcomes related to child length, intelligence and problem-solving, psychomotor development, motor development, and visual acuity among other measures. Most positive effects were dependent upon a specific developmental stage (with some realized short-term at birth to 12 months, and/or medium term at 12 to 24 months, and/or long term beyond 24 months). Lastly, no side effects were reportedly associated with *n*-3 PUFA supplementation [114].

The financial resources of the family can impact maternal consumption of DHA via dietary or supplementary means, ultimately affecting the DHA content of breast milk [115]. Studies of children aged 3–5 years reveal a low intake of ALA, DHA, as well as total fat in low-income countries [116,117]. The intake of fatty acids, especially DHA, of pregnant or lactating women and their young children do not meet the recommendations in many low-income countries [117,118]. Conversely, intake of the *n*-3 PUFA precursor ALA during pregnancy generally meets the recommendation in Mexico and the USA, but does not in Chile, Bangladesh, and India [76,117,119,120]. In studies examining the intake of DHA during pregnancy, none of the women in Mexico, Bangladesh or India met recommendations [76,117,120]. Postnatally, a decline in fat intake from 50% to 25% of energy between birth and 18 months is also observed in children in developing countries, which is primarily driven by a decline in intake of mother’s milk. This decline intensifies further to about 15% of energy from fat by 2–3 years of age [112]. Importantly, the decline in breast milk intake leads to rapid reduction of dietary *n*-3 PUFA intake whereas the *n*-6 PUFA intake remains relatively stable. Since *n*-6 and *n*-3 PUFA precursors compete for the same elongation and desaturation enzymes, this increase in the *n*-6:*n*-3 ratio results in further decreases in *n*-3 PUFAs beyond what is attributable to total ingested levels. These data highlight the need for DHA supplementation in poorer communities in order to promote optimal cognitive development and possibly socioeconomic development.

### 3.4. DHA and Cognition in Children

The effect of DHA supplementation on cognition during childhood is controversial, with studies reporting no effects [121] or suggesting an improvement in verbal learning and memory [122]. Lower total plasma *n*-3 PUFA concentrations in boys of 6–12 years are correlated with a greater number of learning and behavior problems [123]. Children, 7–9 years of age, who were given an *n*-3 PUFA enriched diet for six months exhibited significantly higher plasma and red blood cell DHA and EPA levels, and these PUFA levels correlated with superior verbal learning, spelling and reading abilities [122]. Moreover, activation of the prefrontal cortex, a brain region critical for executive function, during a cognitive challenge was enhanced in 8–10-year-old normal boys who were supplemented daily for eight weeks with 1.2 g DHA compared to placebo [124].

In the DHA Oxford Learning and Behavior (DOLAB) study, Richardson *et al.* performed an RCT in 362 healthy school children aged 7–9 years from mainstream primary schools in The United Kingdom that initially underperformed in reading ability (bottom tertile). The children received either 600 mg of algal DHA each day for 16 weeks or a corn or soybean oil placebo designed to match both taste and color. Reading ability, working memory and behavior were measured using British Ability Scales (BAS II) assessments and parent/teacher observations using the Conners’ Rating Scales. The results showed subtle reading improvements across the sample, but significant results were observed in the poorest-reading subgroup (lowest quintile). This subgroup experienced an eight-month improvement in reading age after DHA supplementation. Moreover, parent-rated behavior problems (ADHD-type symptoms) were significantly reduced by DHA supplementation. These data are valuable because, in contrast to the well-known critical period for the beneficial actions of DHA on brain function during prenatal development and early life, the DOLAB study showed that DHA can affect brain function well beyond early development in healthy children [125].

In a recent Australian RCT, the question was asked whether supplementation of *n*-3 PUFAs or *n*-6 PUFAs could improve cognitive performance in children [126]. A total of 616 term infants were randomized to receive tuna fish oil (high in *n*-3 PUFAs) or sunflower oil (high in *n*-6 PUFAs) from the time breastfeeding ceased or at the age of six months until the age of five years. Academic performance was measured by the National Assessment Program Literacy and Numeracy (NAPLAN) in school years 3, 5, 7 and 9. Plasma *n*-3 PUFA levels were measured at regular intervals until eight years of age. No significant differences in NAPLAN scores were observed between active and control groups. However, at eight years, *n*-3 PUFA levels in plasma were positively associated with the NAPLAN score measured at five years of age. These data are encouraging, because the observed correlation between the academic performance and *n*-3 PUFA levels was evident even though the supplementation had stopped three years earlier. Furthermore, there are a few issues that may explain the lack of overall effect in NAPLAN scale. First, the attrition of the test cohort was extremely high, and less than half of the original study population completed the final assessment. This situation rendered the final test size of the cohort too small to reveal statistical significance. Secondly, a relatively low amount of *n*-3 PUFAs were supplemented (135 mg of DHA and 32 mg of EPA), making it less likely to find significant effects when assessing cognitive performance. Lastly, the authors acknowledged that the parents of the children in the NAPLAN cohort were slightly older and more likely to be tertiary educated. Moreover, the mothers were more likely to have fully breastfed than the mothers of those who did not consent to or provide NAPLAN data, making it very likely that background intake of *n*-3 and *n*-6 PUFAs would also confound the results in this relatively prosperous group [126]. 

The positive effects of a diet rich in DHA on cognitive abilities may also have select sex differences. A large cohort of USA children aged 6–16 years of age, who were part of the third National Health and Nutrition Examination Survey (NHANES III), revealed cognitive benefits of higher *n*-3 PUFA consumption in both boys and girls, but the effects were twice as prominent in girls [127]. The authors speculated that the sex differences were due to a greater need for DHA in girls, since they need it not only for their own growth and development, but also in order to accrue peripheral stores destined to provide DHA for the neurodevelopment of their offspring. Indeed, recent animal work indicates greater peripheral synthesis of DHA from its precursor ALA in females as compared to males, an effect that emerges at the time of sexual maturity [128]. Interestingly, while the peripheral levels of DHA (plasma and liver phospholipids) were higher, the brain levels were similar, leading the authors to conclude similarly to Lassek *et al.* [127] that “the additional production of DHA in the liver of sexually mature females was not particularly intended for the brain, but probably for storage and mobilization upon future pregnancy.”

There is a fair amount of inconsistency between studies examining the effects *n*-3 PUFA supplementation on cognitive performance in children or infants. Thus, there are several potential reasons for varying results including genetic factors, study design and methodological flaws, *etc.* It is also conceivable that genetic variation in fatty acid metabolism affects PUFA status. Potential genetic and environmental factors are not typically considered when recruiting for a study, and they likely contribute to some of the discrepancies between studies. Several study design considerations may help to reduce data variability. For example, stratification of the study population based on baseline DHA levels may help to avoid ceiling effects. It is also worthwhile to specify whether supplements should be taken on an empty stomach or with food, since certain food matrices can affect the bioavailability of *n*-3 PUFAs [129]. Using sensitive, standardized and validated analytical methods and cognitive tests as well as sufficient statistical power, dose and supplementation duration might also help resolve some supplementation effects. Importantly, the source of DHA (the particular type of fish flesh or oil, organ meat, oils derived from algae or crustaceans such as krill, *etc.*) as well as the ratio of EPA to DHA may affect the overall bioavailability of the *n*-3 PUFAs [130,131,132]. Assessment of tissue levels of DHA is also critically dependent upon the sample assayed, where erythrocytes are preferred over plasma fractions since their DHA content correlates better with peripheral and central concentrations of DHA rather than being heavily influenced by recent dietary intake as is the case for plasma [133]. Furthermore, the consumption and tissue levels of other nutrients should be considered. Very recent research published by Jernerén *et al.*, [134] suggests that homocysteine (Hcy) status, which is affected by B-vitamin status, may also determine the effects of *n*-3 PUFAs on cognitive decline and dementia (discussed in a subsequent section). Thus, B-vitamin status may explain some more of the inconsistent data across *n*-3 PUFAs supplementation trials. Last, but not least: it is important that the supplemented materials are of high quality, the source is completely defined, and the dosages are clearly defined and validated. In summary, assessments of dietary intakes of DHA suggest that pregnant and lactating women and young children often do not meet recommendations. Improving the dietary habits of mothers-to-be, pregnant and lactating women, and infants and children, with a particular focus on consuming high-quality sources of DHA, may be feasible strategies to enhance tissue levels and reap the corresponding developmental and cognitive benefits.

### 3.5. Mechanisms of DHA Actions during Development

As already outlined, DHA is accumulated in the brain tissue mainly during the second half of pregnancy and during the first two years of life [135]. Currently the consumption of one to two portions of fish per week, including oily fish, which is a rich source of DHA, is recommended. The challenging question is: What are DHA’s actions in the developing brain that lead to gains in cognitive function? This question is far from being answered, but several pathways can be identified, many of which overlap with those occurring during adulthood and aging (described in a subsequent section).

DHA, as the most abundant *n*-3 PUFA in the brain and retina, contributes to the structure of brain cell membranes. DHA is also implicated in neurogenesis, neurotransmission, and cell survival within the CNS [136,137]. DHA contributes to cell membrane fluidity [138] and to signal transduction within the CNS by activating cell membrane receptors [135]. DHA also alters gene expression in mammalian brain tissue [139,140] that influences neurite outgrowth and learning and memory [141,142]. The process of neurite outgrowth in hippocampal neurons is enhanced by DHA, which may in turn promote learning [141]. Growth of neurites requires the accumulation of lipids in new membranes, and DHA helps to organize lipid raft domains in the membrane by pushing cholesterol into these structures important for neurite extension, myelination, and membrane-mediated signaling [143,144,145,146]. Also important for neurite outgrowth, DHA enhances protein kinase B (PKB; also known as Akt) signaling and in turn the mTOR (mechanistic target of rapamycin) complex, which promotes neuronal growth [147,148]. DHA improves learning and memory by facilitating the formation of pre- and postsynaptic proteins that enable synaptic transmission and long-term potentiation (LTP) [149]. Under oxidative stress, DHA can promote repair and growth of neurons by activating peroxisome proliferator-activated receptor gamma (PPAR) and through its activating effect on syntaxin-3 (STX-3) [150].

DHA is deposited within the cerebral cortex at an accelerated rate during the last trimester of gestation and during the first two years after birth, rendering this phase of neuronal development particularly vulnerable to nutritional insufficiencies [151]. This early accelerated rate of DHA deposition coincides with the onset of myelination, a process that is sensitive to DHA accumulation and stores [54,152]. Notably, animal data show that it is physiologically difficult to reverse the effects of early brain DHA depletion [153] and that reduction of *n*-3 PUFAs in the diet negatively affects DHA concentrations within the brain [154,155,156]. Studies in non-human primates show that low DHA levels are associated with deficits in the visual system, in brain functions [157], and in motor capabilities [158]. Furthermore, animal models provide solid evidence that the consequences of dietary DHA deficiency are a high *n*-6 to *n*-3 PUFA ratio in brain fatty acid composition and deficiencies in learning and memory behaviors [142,159], possibly due, in part, to negative impacts on neurite outgrowth and myelination [160].

## 4. DHA and Cognition in Adulthood and Aging

### 4.1. DHA during Adulthood

Cognitive function reaches its peak during middle adulthood. Thus, the detection of measurable effects of DHA consumption requires lengthy observational or treatment periods, and measurable effects are most effectively observed in cases of DHA-insufficiency or cognitive impairment. Indeed, a recent meta-analysis by Abubakari *et al.* [161] of 12 RCTs in adults found no effect of *n*-3 PUFAs on cognitive measures. However, the authors selected trials with subjects exhibiting a multitude of baseline cognitive conditions and/or subjects diagnosed with psychiatric conditions such as depression and schizophrenia, in addition to cognitive test parameters that likely clouded any measurable effect of *n*-3 PUFAs. Another recent meta-analysis of 34 RCTs found no effect of *n*-3 PUFA supplementation on cognitive performance, but the investigators combined trials performed with children, adults and elderly without performing any subgroup analyses that considered age or dose [162]. This limited their conclusions greatly since assessment of cognitive function is highly dependent on developmental timeline and age. Conversely, a well-designed meta-analysis by Yurko-Mauro and colleagues revealed that DHA + EPA supplementation improves episodic memory outcomes in adults with mild memory complaints, an effect primarily attributable to daily DHA doses above 580 mg [163].

Observational studies suggest a correlation between blood levels of DHA and cognition in healthy adults. For example, higher serum DHA levels were associated with better non-verbal reasoning, mental flexibility, working memory and vocabulary in 35 to 54 year-olds with no neuropsychiatric disorders and no supplemental fish oil use [164]. In an extension of this study, Leckie *et al.* [165] found that a higher ratio of DHA to ARA in serum counteracted the negative effect of low physical activity on both working memory and the trail marking task. This is important since physical activity can increase gray matter volumes and reduce the overall risk for Alzheimer’s disease [166,167].

RCTs performed with adults report mixed results for DHA and *n*-3 PUFA supplementation on cognition. Supplementation of healthy adults from 18 to 70 years of age with 850 mg DHA and 630 mg EPA daily for 12 weeks had no appreciable effect on cognition [168]. Likewise, supplementation of young adults (18 to 35 year-olds) with 1000 mg DHA and 200 mg EPA for 12 weeks had no significant cognitive effects [169]. Furthermore, daily supplementation with 250 mg DHA and 1740 mg EPA in a college-age cohort for four weeks had no effect on attention, memory, or inhibitory responses [170]. In contrast, another study performed with college-age students and a higher daily DHA dose (480 mg DHA and 720 mg EPA) for four weeks found improvements in verbal learning and memory in the supplemented group despite the very short treatment interval [171]. Similarly, Stonehouse *et al.* (2013) reported improvement of episodic and working memory in 18 to 35 year-olds provided a high-DHA supplement containing 1160 mg DHA and 170 mg EPA daily for six months compared to placebo controls [172]. Interestingly, the effect on episodic memory was driven primarily by the women in the study, whereas the men were largely responsible for the effect on working memory. This is not completely surprising given the existence of sex differences in particular cognitive domains, including spatial working memory [173,174]. Recently, it was also shown that deficits in episodic memory observed in lonely individuals was prevented by *n*-3 PUFA supplementation for four months [175]. These data suggest that detecting cognitive effects in adults likely requires substantial levels of DHA provided for extended treatment periods in an experimental cohort with a relatively narrow age range.

### 4.2. DHA during Normal Aging

The age demographics of the global population is shifting as lifespans increase over time due in part to advancements in medicine and positive economic development. The segment of people 65 and older is projected to triple to 1.5 billion globally by 2050 (WHO, [176]). Unfortunately, cognitive ability declines naturally with age even in the healthiest of individuals. This decline is typically subtle, but it is nonetheless undesirable and ultimately affects the quality of life. Environmental factors such as diet, exercise, and DHA consumption can positively affect the normal aging process and overall mental health and performance.

Total gray matter volume declines with age [177], matching a parallel decrease in DHA composition [178]. This drop in DHA may be partially due to changes in activities for the enzymes responsible for DHA accretion into phospholipids [179,180] or to shifts in plasma pharmacokinetics [181]. Importantly, *n*-3 PUFA intake is positively correlated with gray matter volume in adults [182] and in brain regions responsible for cognition in normal, elderly adults [183]. During normal aging there is a gradual 10%–15% loss of total neuronal synapses resulting in a cognitive decline that is typically noticed around age 65 [184], and an increasing risk of dementia that is largely negligible before age 60 [185]. However, processes leading to neuronal loss and the impairment of brain functions may be active at much younger ages [186]. For example, in susceptible young adults, an age-related reduction of neurons in the brain is reported [187]. Of note, declines in episodic memory can begin as early as 20 years of age [188].

Support of the clinical findings above can be found in preclinical animal experimentation, in which age-related neuronal loss was shown to begin at the end of adolescence in rat brains [189]. Total synaptic loss is the best correlate of cognitive decline during aging, as overall synaptic density affects cognitive ability [190]. Increases in oxidative stress and inflammation in both nervous and immune systems also occur with aging [191], leading to DNA damage and telomere shortening. DHA has proven synaptic effects that improve synapse strength and numbers, and DHA can help prevent or mitigate oxidative stress and neuroinflammation. These actions will be discussed in more detail in the section on potential DHA mechanisms during aging.

Many observational studies have linked dietary consumption of *n*-3 PUFAs, and DHA in particular, with improvements in cognitive function and/or reductions in cognitive decline in healthy, aging populations. For example, DHA intake was correlated with performance and speed in a verbal learning test performed in a cohort of 45 to 70 year-old healthy individuals [192]. Van Gelder *et al.* [193] performed a prospective study using longitudinal data that calculated EPA + DHA consumption at baseline and at five years later in 70 to 89 year-olds and found that the decline in baseline mini-mental state exam (MMSE) scores was negatively associated with dietary EPA + DHA levels. In a group of 65 to 80 year-old healthy individuals, consumption of greater than 2.1 g of *n*-3 PUFAs per day was associated with better memory and executive function [194]. Velho *et al.* [195] reported that, in a cohort of healthy elderly (over 65 years-old), those that improved their MMSE scores at 8.5 months from baseline had higher consumption of *n*-3 PUFAs than those who did not show improvements. Cross-sectional analysis of elderly Spanish residents (*N* = 304), with an average age of 75 years-old, found a positive correlation between dietary DHA consumption and MMSE scores and that lower DHA intake was a predictor of cognitive impairment [196]. In a large cohort of Chinese adults (average age of 65; part of the Singapore Longitudinal Aging Studies) the daily consumption of fish oil supplements was associated with higher baseline MMSE scores and a lower risk of decline in cognition over a 1.5-year span [197]. Titiova *et al.* [198] more recently reported a prospective observational study in a healthy elderly population where baseline dietary DHA intake levels at age 70 were positively correlated with larger gray matter volume and declarative memory test performance (seven minute screen) at age 75. In a larger prospective study using data from the China Health and Nutrition Survey, Qin *et al.* [199] examined the decline in global cognitive scores over an average of 5.3 years in a group of Chinese adults (*N* = 1566, mean age 63 years old). This study detected a positive correlation between at least one serving of fish per week and slower declines in global cognitive function, composite and verbal memory scores. Finally, most recently del Brutto *et al.* [200] reported on a cohort from a rural middle-to-low income area of Ecuador that cognitive function as measured by Montreal Cognitive Assessment (MoCA) scores was positively related to the number of fish servings per week, and the data suggested an intake of at least four fish servings per week was best.

While dietary DHA intake is a convenient measure, there are inherent flaws in the reliability and accuracy of data acquired from food frequency questionnaires [201]. Therefore, studies that measure tissue levels of DHA could provide a more appropriate measure of current and past DHA status in relation to cognitive performance. Indeed, some but not all observational studies have linked DHA concentrations in blood with overall cognition and select cognitive domains. For example, a recent study measured the cognitive function of 2157 postmenopausal women at baseline and annually for six years and found no correlation between erythrocyte DHA + EPA and cognitive performance in seven cognitive domains at baseline or over time [202]. On the other hand, the Etude du Vieillissement Arteriel (EVA) cohort of French 63 to 74 year-olds exhibited a reduced risk of overall cognitive decline over four years in those with higher erythrocyte *n*-3 PUFA content [203]. Dullemeijer *et al.* [204] performed a cross-sectional longitudinal study in a Dutch cohort (50–70 years old) and found that higher plasma concentrations of total *n*-3 PUFAs at baseline was associated with less decline in sensorimotor and complex speed-related cognitive domains three years later. However, they did not observe associations with changes in memory, information-processing speed, or word fluency. Whalley *et al.* [205] performed a battery of cognitive tests in a Scottish cohort at ages 64, 66, and 68 years old and found a positive correlation between overall cognitive performance over time and erythrocyte DHA content at baseline (64 years old). Interestingly, this effect was only apparent in the absence of the ApoE4 allele, indicating a genetic influence on the link between peripheral DHA levels and cognition. The Framingham Offspring Study performed in a large group of elderly women (mean age 67 years old) reported that the women with erythrocyte DHA levels (but not EPA) in the lowest quartile had lower brain volume and poorer scores for visual memory, executive function, and abstract thinking than those in the top three quartiles [206]. Lower serum DHA (but not EPA) was also found in a small selected case study group of elderly with reduced performance in the MMSE compared to a cognitively healthy control group [207]. In this study, DHA serum concentrations were correlated with performance on a majority of the tests (memory, attention, and mental flexibility). Recently, Otsuka *et al.* [208] reported results of a cross-sectional longitudinal study in Japanese elderly adults that revealed a link between low serum DHA levels and a greater risk for cognitive decline over a decade as measured by the MMSE. This is especially profound given that elderly Japanese have twice the circulating levels of DHA as compared to similar cohorts in England [208,209]. Overall, these reports provide a suggestive link between the tissue levels of DHA and cognition during aging.

Data from RCTs in healthy aging adults has been historically sparse, but recently more trials have been reported, with some mixed but generally positive effects of DHA and *n*-3 PUFA supplementation on cognition. Initial reports indicated no differences in overall cognition in a group of elderly individuals over 65 years of age receiving daily high or low dose fish oil for 26 weeks, however subgroup analysis did reveal improvements in attention in those with the ApoE4 genotype [210]. Similarly, Dangour *et al.* [209] found no effect of daily *n*-3 PUFA supplementation for two years on cognitive measures in a group of 70 to 79 year old healthy individuals. However, controls in this study did not have a decline in cognitive performance over the two-year span, possibly masking any potential effect of DHA. Additionally, Stough *et al.* [211] reported no effect of daily DHA (252 mg) for 90 days on cognition as measured by Cognitive Drug Research (CDR) scores, however this cohort included a wide range of ages (45 to 80) and a small sample size, making interpretations difficult. In contrast, a combined supplementation of 800 mg DHA and 12 mg lutein for four months in healthy 60 to 80 year-old women, improvements in verbal fluency, memory scores, and rate of learning were seen [212]. 

More recent RCTs have yielded more compelling results than the earlier work. The Memory Improvement With Docosahexaenoic Acid Study (MIDAS) study, performed by Yurko-Mauro and colleagues [213], provided 900 mg of DHA or a placebo daily for 24 weeks in 485 healthy elderly individuals (mean age of 70 years-old) who had a self-reported mild memory complaint and a MMSE score over 26 (cognitively normal). Those supplemented with DHA exhibited improved episodic and visual recognition memory, but not in executive or working memory, and plasma DHA levels were directly correlated to scores in episodic memory. Furthermore, Vakhapova *et al.* [214,215] reported benefits in an elderly population with memory complaints (non-dementia) of improved immediate recall memory and sustained attention when provided a daily 300 mg DHA-phosphatidylserine supplement for 15 weeks. In a cross-over RCT, Nilsson *et al.* [216] reported a benefit of 3 g daily *n*-3 PUFA supplementation (1050 mg DHA) for five weeks in measures of working memory and selective attention in a group of middle age to elderly subjects. It is intriguing to think that the metric used by Yurko-Mauro *et al.* and Vakhapova *et al.* of a mild subjective memory complaint might be an early correlate of age-related cognitive decline, and their findings could indicate the potential power of DHA supplementation during the long, early and undetectable phases of cognitive impairment and dementia. 

Recent RCTs have also associated the cognitive benefits of DHA with neurophysiology or anatomical changes in the brain. Witte *et al.* provided *n*-3 PUFA supplementation (880 mg DHA and 1320 mg EPA) for 26 weeks in healthy subjects (age 50 to 75) and found improvements in executive function, white matter integrity, gray matter volume, and parameters of neurovascular function compared to individuals provided a placebo. Tokuda *et al.* [217] provided 55 to 64 year old Japanese men who already consumed an average of 543 mg DHA per day and had substantial plasma DHA levels (7.0% of fatty acids) a supplement containing 300 mg DHA, 100 mg EPA, and 120 mg of ARA daily for four weeks. They report that treatment prevented a decline in auditory event-related potential (ERP) latencies (a measure of cognitive processing speed) that was observed in the placebo group. Deficits in auditory ERPs are typically observed in Alzheimer’s disease [218]. Finally, a very recent study reported by Strike *et al.* [219] suggests that a combined supplemental approach may be beneficial. They gave a group of postmenopausal women between the ages of 60 and 84 years-old a daily supplement containing 1000 mg DHA, 160 mg EPA, 240 mg ginkgo biloba, 60 mg phosphatidylserine, 20 mg tocopherol, 1 mg folic acid, and 20 g vitamin B12 for six months and found a shorter mean psychomotor response latency (a measure of information processing speed) and verbal recognition memory in the treatment group relative to the placebo group. They did not detect any changes in executive function or paired associate learning. In totality, data from studies reporting the effect of dietary DHA consumption, blood DHA concentrations, and supplementation with DHA on parameters of cognition in normal aging individuals provide a substantial argument for obtaining sufficient amounts of DHA via dietary or supplemental means during aging.

### 4.3. DHA and Cognition in Mild Cognitive Impairment and Dementia

During aging, an increasing share of the resources available for normal cellular maintenance are spent on repair mechanisms needed by the cell to cope with the cumulative effects of oxidative, inflammatory, and other environmental insults. With this shift in demand, any diminished availability of energy, as a result of poor mitochondrial function, may result in neurodegenerative processes that can lead to neuronal loss and eventually to cognitive impairment, dementia, or other neuropsychiatric maladies. Unfortunately, neurodegenerative processes and neuronal cell death occur well before clinical signs of cognitive deficits are confirmed [186]. Dementia is not a disease itself, but rather a group of chronic symptoms that are common to several neuropsychiatric disorders (Alzheimer’s disease (AD), Lewy body dementia, Parkinson’s disease, *etc.*). Dementia symptoms, such as deficits in memory, language and executive function, lead to poor cognitive function in these individuals [186]. Dementia often results in poor self-care that can lead to inadequate nutrition, which could potentially exacerbate the cognitive deficits. In the most severe cases of dementia there is a loss of functional independence that results in institutionalization. Worst-case scenarios occur in low- and middle-income, developing countries where access to nutrient-dense foods and the ability to afford medical and functional care are leading to exponential growth in dementia prevalence rates [220].

There is a normal degradation in cognitive ability and brain atrophy with age [221,222]. However, the rate of atrophy is markedly higher in mild cognitive impairment (MCI) and dementia. The atrophy rate is especially high in the subgroup of MCI subjects that eventually develop clinically diagnosed AD [223], the most prevalent neurodegenerative disease. Approximately half of all individuals with MCI progress into AD within five years. Furthermore, brain glucose metabolism decreases 10%–15% during normal aging, and the extent of cognitive decline in MCI and AD is associated with the degree of glucose metabolism loss (nearly 35% in some brain regions; [224,225,226]). Preclinical animal studies have convincingly shown that DHA provided over a substantial amount of time can reduce neuronal loss and improve learning and memory as the animals age (For meta-analysis, see [227]). However, studying the effects of DHA on the risk of dementia is very challenging given the low incidence rates in cognitively healthy individuals. Some estimates have called for nearly 50,000 participants at baseline for proper statistical power [228], whereas the rate of cognitive decline during dementia is a more accessible measure. In addition, changes in cognitive domains are dependent upon the specific type of dementia. For example, early changes in AD are seen in episodic memory, whereas in vascular dementia early deficits in executive function are seen. Later stages of dementia and AD involve a multitude of cognitive domains.

Observational studies have linked the consumption of *n*-3 PUFAs with a lower prevalence of dementia [229] and lower overall risk of developing dementia [230]. Albanese *et al.* found a significant dose-dependent decrease in dementia relative to fish intake in a large group of subjects (14,960) residing in middle-to-low income areas [229]. The Three-City cohort study of 8085 French residents over the age of 65 found an inverse relationship between fish consumption and overall risk of dementia over a four-year timeframe in ApoE4 non-carriers (80% of subjects) [230]. Blood levels of DHA have also been tied inversely to mild cognitive impairment (MCI) and dementia. For instance, in the Framingham study, plasma phosphatidylcholine fatty acid content was measured in 899 subjects with an average age of 76 and no dementia at baseline. When re-assessed for cognitive ability nine years later, individuals in the top quartile for baseline plasma PC-DHA levels had a 47% lower risk of all-dementia (grouped with AD) *versus* the other three quartiles combined. No other fatty acid was significantly correlated (including EPA), and food intake surveys revealed that this quartile had an average DHA intake of 180 mg per day [231,232]. Cherubini *et al.* [233] also reported higher levels of plasma DHA in cognitively normal subjects as compared to those with dementia in an aging Italian cohort. With regards to MCI, Milte *et al.* [234] detected higher levels of the *n*-6 PUFA docosapentaenoic acid (DPA*n*-6; 22:5(*n*-6)) in the erythrocytes of MCI patients relative to healthy controls. More recently, Yin *et al.* [235] reported lower blood levels of DHA in amnestic and multi-domain MCI patients as compared to normal control subjects. These are intriguing findings since the DPA*n*-6 replaces DHA in the brain during DHA deficiency in an inefficient attempt to retain function.

Combinations with other nutrients are likely also important. Interesting results from the homocysteine and B vitamins in cognitive impairment (VITACOG) trial were very recently published in which 168 patients with MCI (≥70 years of age) were given placebo or high dose Hcy-lowering B vitamins (folic acid, B6, B12) and assessed for brain atrophy via MRI at baseline and at a two-year follow-up [134]. B vitamin treatment reduced the brain atrophy rates by 40%, an effect only observed in the subgroup with the highest tertile of baseline plasma *n*-3 PUFA levels. Baseline plasma DHA, but not EPA, was a significant predictor of reduced yearly brain atrophy rate in those who took B vitamins, but not in placebo controls. Thus, the DHA status of the MCI patient affected the correlation between vitamin B supplementation and brain atrophy, and possibly cognitive decline by extension. It would be interesting to determine whether the Hcy and DHA status of the MCI subject affects or predicts the rate of conversion from MCI to dementia (about 5%–10% per year). If so, perhaps supplementation with B vitamins in combination with DHA in MCI patients would be an effective prophylactic treatment aimed at reducing the risk of further cognitive decline and the development of dementia.

RCTs have provided evidence indicating largely positive effects of *n*-3 PUFA supplementation on cognitive measures in subjects with MCI or dementia. Early evidence from Terano *et al.* [236] indicated improved dementia scores in patients with moderately severe dementia caused by thrombotic cerebrovascular disorder who received 720 mg of DHA daily for 12 months. Combined supplementation with DHA (240 mg) and ARA (240 mg) daily for 90 days was shown to improve attention and immediate memory in patients with mild cognitive dysfunction [237]. Furthermore, supplementation with 720 mg DHA and 1080 mg EPA daily for 24 weeks in MCI patients improved their scores in the Clinician’s Global Impression of Change (CIBC)-plus and Alzheimer’s Disease Assessment Scale (ADAS)-cog [238]. Sinn *et al.* [239] administered several doses of *n*-3 PUFAs, including high EPA (1670 mg), high DHA (1550 mg), or high LA (2200 mg) daily for six months in MCI patients and detected improvements only in the high DHA group particularly for Initial Letter Fluency, a measure of fluid thinking ability. The other cognitive measures did not show any differences, but the baseline erythrocyte DHA levels in this study were higher than those of Chiu *et al.* (5% *vs.* 4.2%). In a small, preliminary trial of 25 MCI patients, administration of a high-DHA (1440 mg) supplement also containing small amounts of EPA, tryptophan, phospholipids, and melatonin daily for three months improved MMSE scores, semantic verbal fluency and olfactory sensitivity [240]. The benefits of DHA on cognition in MCI may be dose-dependent since a recent report found no effect of daily supplementation with 180 mg DHA plus 120 mg EPA for 180 days in mild to moderate MCI patients on scores in the MMSE and Abbreviated Mental Test (AMT) [241]. Overall, optimal tissue levels of DHA are important in reducing the likelihood of developing, and improving the symptoms of, MCI and dementia.

### 4.4. DHA and Cognition in Alzheimer’s Disease

AD is a uniquely human, progressive neurological disease resulting in hallmark neuropathology consisting of senile plaques, neurofibrillary tangles, neuronal atrophy, and abnormal brain glucose metabolism. AD accounts for more than 70% of dementia cases and has an estimated worldwide prevalence of about 4.4% of the population over 65 years old [242]. This prevalence is expected to grow from 5.2 million Americans in 2014 to 13.8 million Americans and to 115 million people globally by 2050 (Alzheimer’s Association 2014 report [243], WHO 2012 Dementia Report [244]). Deaths from AD rose by 66% in a recent eight-year span, highlighting the lack of efficacious therapeutic options currently available, aging societal demographics, and shifting environmental impacts such as nutrition and physical activity [186]. A recent study in Medicare fee-for-service beneficiaries in the US found that the average total cost for a patient with dementia during the final five years of life was nearly $300,000. The out-of-pocket expenses during the final five years of life were approximately 80 times more for dementia patients when compared to those with heart disease or cancer [245]. Caretaking costs associated with dementia and Alzheimer’s in the US is not generally reimbursed by Medicare, and these costs often completely deplete the household wealth of the patient and/or family member caretakers. Unfortunately, no therapeutic cure is currently available, and few investigational new drugs are currently being tested as pharmaceutical companies have traditionally experienced frequent failures in attempts to find efficacy [186,246].

Late onset sporadic AD (LOAD) is the most prevalent form of AD and has the lowest identifiable link to genetics. Therefore, LOAD may be most sensitive to environmental factors, such as diet and DHA intake. This is especially true given that neuropathological changes occur decades before clinically identifiable cognitive deficits, providing a long window of time for the cumulative effects of environmental factors to affect the manifestation of the disease. Traditionally, clinical studies have focused on later stages of the disease, when the disease neuropathology appears to be intractable and resistant to therapeutic approaches. AD affects 32% of people over 85 years old [247], and age is the single greatest risk factor, suggesting that even AD can be considered normal physiological aging.

Brain DHA composition likely plays a role in AD. The brains of non-DHA supplemented Alzheimer’s patients have 65–95 nmol/g of unesterified DHA, much less than normal controls (110 nmol/g; [248,249]). Furthermore, deficient liver biosynthesis of DHA has been observed in AD patients, where it appears that DHA biosynthesis halts at the last β-oxidation step from tetracosahexaenoic acid (24:6*n*-3) to DHA [248]. However, this is likely a minimal source of DHA (*vs*. preformed via diet). Recent data also suggests that AD patients have problems processing DHA [61], therefore the magnitude of DHA’s effects may be less in later stages of the disease [250]. Phospholipids PC and PE from various brain regions (particularly hippocampus) in the AD brain have reduced DHA content compared to control brains, further implicating DHA in the etiology of the disease [251,252].

Observational studies are generally supportive of a preventative role of dietary *n*-3 PUFAs and DHA with regards to risk and incidence of AD. Morris *et al.* [253] found that one or more servings of fish per week (or about 200 mg of DHA) was associated with a 60% lower risk of developing AD and that total intake of DHA (but not EPA) was also a determinant of lower AD risk. Furthermore, patients with early stage AD reportedly have lower dietary intakes of *n*-3 PUFAs as compared to healthy individuals [254]. Very recently, the AD Neuroimaging Initiative trial reported results in 229 normal, 397 MCI, and 193 AD patients assessed frequently over a two-year period. They found significant correlations between fish oil supplement use and lower brain atrophy in the hippocampus and cortical gray matter areas across all subjects [255]. There is some inconsistency in the reports of blood DHA levels in AD patients likely due to altered DHA pharmacology and bioavailability and the particular tissue or lipid fraction analyzed. One of the earlier reports indicated that total PL, PC and PE isolated from the plasma of subjects with AD, dementia, or cognitive impairment no dementia (CIND) contained less DHA than found in healthy elderly controls [256]. Furthermore, Tully *et al.* [257] reported that community-living elderly with AD had approximately half the serum concentrations of cholesteryl ester-DHA in comparison to non-dementia controls. These data also indicated that DHA and total saturated fatty acid levels were determinants of the clinical dementia rating. Wang *et al.* [258] found that lower scores on the MMSE in mild and moderate AD patients were associated with lower erythrocyte DHA content. More recently, Lopez *et al.* [259] evaluated an elderly cohort with an average age of 80 years-old for dementia and blood DHA levels. They reported that plasma DHA levels in the highest tertile had 65% reduced odds of all-cause dementia and a 60% reduced chance of AD, an effect that was recapitulated with dietary intake questionnaire data (highest tertile of DHA intake had 72% reduced odds of developing AD). Phillips *et al.* [260] also found positive correlations between composite memory scores (verbal reasoning, contextual, visual, and verbal memory) and overall cognitive status with plasma PC-DHA content across a cohort of normal elderly, cognitive impairment no dementia, and AD subjects.

RCTs investigating the therapeutic potential of DHA in improving the symptoms of AD are scarce and have mixed results at best. One of the first studies was performed by Kotani and colleagues [237], where they supplemented AD patients for 90 days with a daily dose of 240 mg DHA plus 240 mg ARA. This study found no significant changes in the repeatable battery for assessment of neuropsychological status test (RBANS; test five main cognitive domains). It would have been intriguing if Kotani *et al.* had a DHA-alone group to assess any potential counteractive effects of ARA in the combination administration. Subsequently, initial results from the OmegAD study in 174 mild-to-moderate AD patients (mean age 74) provided 1720 mg DHA plus 600 mg EPA daily for six months found no changes in ADAS-cog, MMSE, or Alzheimer’s Prevention Initiative (API) tests [261,262]. However, in this early report of the OmegAD study, they did discover that a small subset of AD patients with milder cognitive dysfunction had lower declines in MMSE scores after supplementation. The next reported study was a small preliminary RCT that dosed AD patients for 24 weeks with a daily supplement containing 720 mg of DHA and 1080 mg of EPA. It reported improvements in the Clinician’s Interview-Based Impression of Change Scale (CIBIC-plus), but not the ADAS-cog score [238]. Subsequently a large (*N* = 295), multi-center (51) study was reported by Quinn and colleagues [250], where they provided approximately 1000 mg of algal DHA daily for 18 months to patients with mild to moderate AD. They did not detect any differences in ADAS-cog or clinical dementia rating, suggesting that DHA is likely more effective as a prophylactic rather than a therapeutic treatment for AD. Interestingly, DHA-treated subjects in the ApoE4 negative subgroup had less decline in ADAS-cog and MMSE over time *versus* placebo, indicating a potential genotype dependence for DHA’s effects in AD. Most recently, updated results have been described by the investigators of the OmegAD study where they have analyzed the levels of *n*-3 PUFAs present in the plasma acquired at baseline and after six months of the high-DHA supplement [263]. They report that increasing plasma levels of DHA in these AD patients was tied to preservation of cognition as measured by ADAS-cog scores. Higher concentrations of plasma DHA resulted in a lower rate of cognitive decline, an effect that was similar across genders. Cerebrospinal fluid (CSF) measures were also obtained in a small group of patients (*N* = 33) from the OmegAD study [264]. The patients in the treatment group had significant increases in the concentration of DHA in the CSF, and these concentrations were inversely correlated to CSF levels of tau (total and phosphorylated) and directly proportional to CSF levels of interleukin (IL)-1 receptor type II (anti-inflammatory effect). Tau levels are elevated during the prodromal phase (total tau) and clinical phase (phosphorylated tau) of AD, and these intriguing results suggest that DHA may be able to mitigate this increase to some extent.

The role of DHA in aging and dementia continues to be an emerging area of research, and more clinical work is certainly warranted given the limited, yet promising results thus far. The later stages of AD are largely intractable, so emphasis should be placed on prophylactic and early-stage therapeutic uses of DHA. Recent negative RCTs in AD patients have been focused on symptomatic effects in already diseased individuals, where significant and irreversible neuronal loss has occurred. DHA effects may be best determined with the disease-modifying effects of DHA focused upon in a large secondary population with mild cognitive complaints (much like the MIDAS study), along with several risk factors for dementia and AD (e.g., ApoE4, cardiovascular disease, low *n*-3 PUFA levels, early plasma AD biomarkers, etc). Pre-planned analysis of DHA effects within specific subgroups (dementia, genotype, baseline indicators, *etc.*) on repeated measures of cognitive function over a substantial amount of time is preferable, but this approach would certainly be costly and time-consuming. Nonetheless, these types of trials are needed to determine the prophylactic effects of DHA and the population subgroups that may benefit the most. In this regard, the ability of DHA to save neurons, and ultimately cognitive capacity, from a seemingly predestined fate could be determined. Considering that the aging of the brain occurs over decades, a general recommendation for maintaining DHA consumption throughout adulthood seems appropriate given the substantial data in aging populations, regardless of cognitive ability or disability.

### 4.5. Mechanisms of DHA Actions during Aging

Many of the effects of DHA during aging likely occur via several of the same pathways utilized during development. These include those important for neuron growth and survival, maintenance of myelination, reduction or resolution of inflammation, synaptic plasticity, membrane receptor function and lipid raft organization. There is certainly overlap with mechanisms of DHA action between normal and pathological aging. For example, the human brain contains extensive myelination that requires resource-demanding maintenance and repair, and oligodendrocytes, the primary myelinating cell of the CNS, require 2–3 times more energy other brain cell types [265]. It is this homeostatic maintenance and repair of myelin that some researchers consider the weakest link in maintaining brain health in the face of environmental insults and undesirable genetic factors. Myelin sheath abnormalities and defects are observed in normal aging, and white matter is affected in the initial stages of neurodegeneration [266]. Additionally, brain regions myelinated last in development are first to be affected in AD [267], suggesting a unique susceptibility of these late-myelinated areas to white matter defects and degeneration [268]. These observations have led to the hypothesis that amyloidogenesis and hyperphosphorylation of tau might be byproducts of pathological strain on myelin homeostatic processes, rather than causes [269]. The brain sacrifices axonal transport mechanisms for the sake of saving myelin and/or as a result of poor glucose uptake and overwhelmed anti-oxidative capacity of the neuron. These circumstances often result in synaptic loss and axonal degeneration [269,270]. Interestingly, Virtanen *et al.* [271] found that, in a large cohort of elderly subjects (*N* = 3660; ≥65 years old), higher plasma DHA was associated with a better white matter grade as measured by MRI as well as a lower risk of subclinical brain infarcts. White matter intensities are a predictor of AD conversion in MCI subjects, and silent brain infarcts are associated with more precipitous declines in cognitive functions over time [272].

DHA also likely has cardiovascular benefits throughout adulthood leading to better perfusion of the brain. These benefits include lower blood pressure, improved vasoreactivity, dampened hepatic triglyceride synthesis, and reduced platelet aggregation. The brain is decidedly the most perfused organ of the human body [273], and cardiovascular diseases (common with aging) increase the risk of developing dementia [274]. Vascular dementia is the second most common form of dementia behind AD, and cerebral blood flow reductions and vascular pathologies are often reported in AD [275,276]. Jackson and colleagues [277] provided young adults (mean age of 22 years old) DHA-rich oil, EPA-rich oil, or placebo daily for 12 weeks and reported increased cerebral blood flow as measured by concentration of oxygenated hemoglobin in the DHA group as compared to placebo controls. There was no effect of high-EPA oil treatment on blood flow. This is in line with results from Beydoun *et al.* [278], who observed a positive correlation between *n*-3 PUFAs levels in plasma and cognitive performance in hypertensive and dyslipidemic middle-aged people. Moreover, this correlation was stronger than what was observed in healthy individuals. In addition, Baierle *et al.* [207] found that both increased blood Hcy levels (a risk factor for cardiovascular disease) and reduced serum DHA levels were associated with decreased cognitive performance in healthy elderly. DHA can alter the expression of genes encoding enzymes important for Hcy metabolism, methionine adenosyltransferase (MAT) and methylenetetrahydrofolate reductase (MTHFR) [279], and plasma Hcy and MTHFR polymorphisms are risk factors for dementia [280].

DHA can also affect processes involved in neural plasticity and LTP, which are essential for proper learning and memory function. DHA increases brain-derived neurotrophic factor (BDNF) and both DHA and BDNF affect AkT and ERK/MAPK/CREB signaling pathways to ultimately promote neural plasticity and LTP, which require synaptic modifications that are critical to learning and memory [281,282,283]. Calcium/calmodulin-dependent kinase II (CaMKII) and *N*-methyl-d-aspartate (NMDA) receptor function are essential for the maintenance of LTP and are positively modulated by DHA [284,285]. DHA also mitigates loss of the intracellular scaffold in neurodegeneration models, helping to maintain healthy axons and synaptic structures integral to cognitive function [284,286]. Furthermore, DHA supplementation may improve cognition by enhancing neurogenesis via the retinoid X receptor (RXR) and retinoic acid receptor (RAR), which decrease in expression with age in animal models [287].

The aging of the brain, and especially pathological aging, is also considered to be a consequence of sustained chronic inflammation. Alzheimer patients replete with amyloid plaques who do not exhibit signs of dementia (high pathology controls) have very few signs of neuroinflammation and neurodegeneration [288,289,290], suggesting that an inflammatory component to AD is at minimum partially responsible for the neurodegeneration and dementia. Brains from AD patients exhibit high levels of activated microglia [291], and the degree of cognitive impairment is inversely correlated with the extent of microglial activation [292,293]. Cytokines from these activated microglia break down neural membranes and release pro-inflammatory ARA metabolites, and anti-inflammatory DHA metabolites. Elevated pro-inflammatory cytokines have been identified in the CSF of AD patients [294,295,296], and amyloid beta (Aβ) itself has been shown to induce pro-inflammatory cytokines via activation of inflammasomes [297,298]. DHA can potentially counteract the effects of Aβ since recent data indicates that DHA enhances phagocytosis of Aβ42 by human microglia [299]. Further implicating inflammation in neurodegeneration, gestational immune insult via a viral mimetic, followed by a second similar insult in adulthood, leads to an AD-like phenotype in mice that includes CNS protein aggregates and deficits in cognition [300]. Interestingly, our recent work in this maternal immune activation model has shown that DHA provided throughout development and adulthood significantly dampens subsequent immune stimulation by a second insult with a viral mimetic in adulthood [301].

Important to the resolving process of inflammation, specialized pro-resolving mediators (SPMs) are derivatives of *n*-3 PUFAs and are reduced in AD where brain inflammation is increased [302]. SPMs have anti-inflammatory and pro-resolving characteristics and include protectins, D-series resolvins, and maresins derived from DHA via cyclooxygenase (COX) and lipoxygenase (LOX) pathways [303]. Recent results from the OmegAD trial found that cultured peripheral blood mononuclear cells (PBMCs) from AD patients who received the *n*-3 PUFA supplementation maintained levels of SPMs lipoxin A4 and resolvin D1 in culture over time, even when insulted with Aβ40, whereas PBMCs from control AD patients did not [302]. Furthermore, the effect on SPMs was positively correlated with cognitive changes and changes in transthyretin (prealbumin), which has been shown to inhibit the toxic effects of A. DHA and its metabolites can affect inflammation via several pathways including activation of PPARs, inhibition of nuclear factor kappa-B (NFκB), and activation of the transmembrane receptor GPR120. This is confirmed by observational studies showing that blood levels of *n*-3 PUFAs are associated with lower cytokine levels [304,305], and supplementation with fish oil for 26 weeks changes over 1000 genes in the PBMCs of an elderly cohort, resulting in a more anti-inflammatory gene expression profile [306]. Furthermore, daily supplementation with high-DHA (1700 mg DHA and 600 mg EPA) for six months in AD patients (OmegAD study) reduced the levels of lipopolysaccharide (LPS)-induced cytokines (IL-1B, IL-6) released from isolated PBMCs relative to within-patient baseline levels [307].

During pathological aging of the brain (and even in some cognitively normal elderly), inflammation results in neurofibrillary tangles made of abnormal forms of tau protein that cause alterations in cytoskeletal stability, axonal transport, and loss of synaptic contacts [269,300]. This can affect protein extrusion mechanisms and axonal energy metabolism, resulting in even more phosphorylated tau (p-tau), axonal blockage and leakage, and ultimately cell death. DHA can reduce p-tau levels likely by inhibition of the phosphorylation of tau via c-Jun *N*-terminal kinase 1 (JNK1) [308,309] or Akt through glycogen synthase kinase-3β (GSK3β) [308,310], and help to stabilize white matter [311]. Activation of the Akt pathway promotes cell survival via inhibition of caspase-3, potentially saving neurons during metabolic stress in neurodegeneration. These effects are similar to those seen in developmental reelin-signaling pathways that affect neuronal migration and cortical structure [312,313]. Reelin expression decreases with age and increases with sufficient DHA, and reelin is thought to be involved in the pathogenesis of AD [314]. Both reelin and DHA increase phosphoinositide 3-kinase (PI3K) activity, which activates AkT, which in turn inhibits GSK3β. GSK3β inhibits glycogen production and phosphorylates tau, and ultimately it is this poor glucose uptake and storage in combination with tau hyperphosphorylation that likely precipitates AD pathology [315]. This is in line with emerging data that suggest AD pathophysiology is at least partially mediated by impairments in brain insulin sensitivity, and in glucose metabolism and utilization, that lead to oxidative stress and inflammation [316]. Accordingly, diabetes patients are more likely to develop dementia [317]. Interestingly, animal and *in vitro* studies identify positive effects of DHA on endothelial and glial GLUT1 levels and brain glucose uptake [318,319,320,321].

In AD, extraneuronal Aβ aggregates to form senile plaques that limit plasticity and spine formation, promote loss of memory, increase inflammation via activated microglia, and increase pro-inflammatory cytokines. DHA can inhibit the activity of β-secretase, thereby inhibiting the formation of aggregates [322], and stimulate microglia to phagocytose Aβ peptides *in vitro* [299]. The production of Aβ from amyloid precursor protein (APP) via β-secretase is impacted by the integrity of lipid rafts, which can act to separate APP from the enzyme. Interestingly, there is a decrease in the DHA content of lipid rafts in cortex of AD patients [323], and this decrease in DHA enhances interactions between APP and β-secretase, thereby promoting amyloidogenesis in these subjects [324]. Furthermore, Aβ42 interacts with caveolin-1 containing membranes of erythrocytes in a stronger fashion in DHA-enriched erythrocytes, suggesting that DHA might help with the clearance of Aβ via lipid-raft mediated degradation [325]. These data are corroborated by a cross-sectional study that measured plasma Aβ40 and Aβ42 in 1,219 normal elderly over 65 years of age and found a strong inverse correlation between *n*-3 PUFA intake and plasma Aβ40 and Aβ42 levels [326].

## 5. General Considerations and Conclusions

There is substantial evidence regarding DHA’s importance during pregnancy and infancy on the development of the brain and resulting cognitive function of the child. These effects on learning ability are dependent upon accumulation of DHA during gestation and nursing, and they highlight the need for maternal consumption of dietary or supplemental sources of DHA. Beyond development, a greater understanding of the impact of DHA across decades of life may require piecing together several well-designed longitudinal epidemiological studies. However, epidemiological studies may underestimate the effect of DHA on cognition due to other long-term factors that impact mortality and therefore those potentially helped most by DHA do not reach the age at which cognitive decline or dementia occurs. Nutrition and diet can also be affected by education and socioeconomic factors that can influence cognitive abilities, not to mention a propensity for a healthier lifestyle in general. Such long-term effects on overall health, and in particular on the cardiovascular and neurological systems, will clearly play into an individual’s cognitive abilities.

Regarding intervention trials, inter-individual and day-to-day intra-individual variability in DHA consumption likely contributes to an under-estimation of the effect of DHA supplementation on cognition in RCTs. The interventions are comparably much shorter than long-term dietary habits, and RCTs are often not focused on a subject pool that is likely to benefit the most from interventions, such as those with insufficient DHA levels. Accordingly, basal DHA levels must be taken into consideration, as an effect of DHA is likely to be inconsequential in those with adequate tissue levels of DHA. Linking familial risks with early plasma indicators of AD and dementia (C-reactive protein, interleukin-6 and α1-antichymotrypsin, for example) with plasma DHA status may also help to identify those with the most potential for benefit from supplementation. In observational trials, caution must be exercised when examining dietary DHA intake levels and blood concentrations of DHA. Dietary intake only accounts for 66% of the variance in erythrocyte *n*-3 PUFAs concentrations, indicating that age, sex, BMI, physical activity, micronutrient status and other factors are also involved [327]. Similarly, due to the long half-life of brain DHA (2–3 years), fatty acid content of blood compartments may not accurately depict brain lipid composition, especially in the cases of subjects with cognitive impairment or AD and their likely associations with altered DHA processing [61,328]. Even if peripheral levels are comparable between subjects, the availability of DHA for various tissues and cell types may very well be different. This is highlighted by some recent studies utilizing isotopically labeled DHA indicating changes in DHA dynamics within the body as we age. DHA dynamics may also be dependent on genetic factors such as ApoE4 allele carrier status. For example, a single oral 50 mg dose of ^13^C-DHA took significantly longer to clear from the blood of elderly subjects than young subjects [181]. Furthermore, the half-life of ^13^C-DHA has been measured as 32 days in ApoE4 carriers, and 140 days in non-carriers [329].

Another consideration is the use of other supplements in boosting the effects of DHA, or acting in synergy, especially with regards to aging. For example, curcumin can boost DHA levels in the brain of animals [330], and it is intriguing to speculate that curcumin could help mitigate the losses in DHA that are associated with the heightened β-oxidation that is observed in AD. As previously mentioned, B vitamins and DHA work in tandem to reduce brain atrophy in MCI patients [134]. B vitamins lower Hcy, an intermediate in some oxidative stress-related pathways that are a risk factor for vascular disease as well as dementia. Hcy can also serve as notification that pathological neurodegenerative processes are occurring. Additionally, B vitamins may promote DHA incorporation into phospholipids, and likely have synergistic anti-inflammatory effects. In support of a combinatorial approach, Scheltens *et al.* [331] observed improvements in memory after daily supplementation with DHA, EPA, phospholipids, choline, uridine monophosphate, vitamin E, vitamin C, selenium, folic acid, vitamin B6, and vitamin B12 for 24 weeks in subjects with mild AD.

The non-steroidal anti-inflammatory drug (NSAID) aspirin might be another potential synergistic agent. Acetylation by aspirin enables COX-2 to initiate the biosynthetic pathway that produces resolvins (D1–D6) from DHA [332,333]. Aspirin-dependent resolvins are potent DHA-derived pro-resolving immune mediators [334]. Resolvin D3 has been shown to be especially effective in the late-resolving phase of inflammation (catabasis), the completion of which is critical to prevent an acute inflammatory response from becoming chronic activation [335]. The use of NSAIDs has been associated with a reduced risk for AD, especially in long-time users [336,337]. However, these effects are not realized in currently diagnosed AD, highlighting the likely importance of preventative supplementation [338].

Overall, DHA appears to have the ability to influence many different signaling pathways, receptor systems, enzyme activities, membrane structures and dynamics that ultimately lead to overall better development, maintenance and aging of the CNS, resulting in optimal cognition throughout the lifespan. These benefits likely require a sustained supply of DHA across development, adolescence and adulthood to build and maintain sufficient pools and/or to replenish depleted neural stores. For those unable to obtain sufficient amounts of DHA via dietary means, supplemental DHA from fish oil or vegetarian (algal oil) sources is ideal. DHA-containing supplements are taken daily by millions of people worldwide and have been shown to be safe and well tolerated even at high doses [339].

## Abbreviations

The following abbreviations are used in this manuscript:

Aβ: amyloid beta
AD: Alzheimer’s disease
ADAS: Alzheimer’s disease assessment scale
Akt: protein kinase B (also PKB)
ALA: α-linolenic acid
AMT: abbreviated mental test
API: Alzheimer’s prevention initiative
ApoE: apolipoprotein E
APP: amyloid precursor protein
BBB: blood brain barrier
BDNF: brain-derived neurotrophic factor
CaMKII: calcium/calmodulin-dependent kinase II
CDR: cognitive drug research
CIBC: clinician’s global impression of change
CIND: cognitive impairment no dementia
COX: cyclooxygenase
CNS: central nervous system
CSF: cerebrospinal fluid
DHA: docosahexaenoic acid
DHA-CoA: docosahexaenoic acid coenzyme A
DPAn3: omega-3 docosapentaenoic acid
DPAn6: omega-6 docosapentaenoic acid
EPA: eicosapentaenoic acid
ER: endoplasmic reticulum
ERP: event-related potential
FABP: fatty acid binding protein
FFA: free fatty acid
GSK3β: glycogen synthase kinase 3-β
Hcy: homocysteine
IL: interleukin
IQ: intelligence quotient
JNK1: c-Jun *N*-terminal kinase 1
LA: linoleic acid
LDL: low density lipoprotein
LOX: lipoxygenase
LPS: lipopolysaccharide
LTP: long-term potentiation
MAT: methionine adenosyltransferase
MCI: mild cognitive impairment
MMSE: mini-mental state exam
MoCA: montreal cognitive assessment
MTHFR: methylenetetrahydrofolate reductase
mTOR: mechanistic target of rapamycin
*n*-3: omega-3
NFκB: nuclear factor kappa-B
NMDA: *N*-methyl-d-aspartate
NSAID: non-steroidal anti-inflammatory drug
PBMC: peripheral blood mononuclear cells
PC: phosphatidylcholine
PE: phosphatidylethanolamine
PI3K: phosphoinositide 3-kinase
PL: phospholipid
PPAR: peroxisome proliferator-activated receptor gamma
PUFA: polyunsaturated fatty acid
PS: phosphatidylserine
RAR: retinoic acid receptor
RBANS: repeatable battery for assessment of neuropsychological status test
RCT: randomized control trials
RXR: retinoid X receptor
SPM: specialized proresolving mediators
STX-3: syntaxin-3
TAG: triacylglyceride
